# Occult Cervical Avulsion: A Rare Cause of Intrapartum Vaginal Bleeding

**DOI:** 10.1155/2019/7356150

**Published:** 2019-04-30

**Authors:** Charity Hulin, Brennan Lang, Jennifer Stanley, Christina Davidson

**Affiliations:** Department of Obstetrics and Gynecology, Baylor College of Medicine, Houston, TX, USA

## Abstract

**Background:**

Cervical avulsion, complete or partial, is a rare intrapartum complication that can go unrecognized without proper physician vigilance.

**Cases:**

Case 1 is a 32-year-old multiparous woman admitted for induction of labor at 37 2/7 weeks for abnormal antenatal testing. Case 2 is a 22-year-old multiparous woman at 29 0/7 with spontaneous preterm labor 10 days after cerclage removal for preterm contractions. Vaginal bleeding and fetal heart rate decelerations complicated each patient's intrapartum course. Annular cervical detachment noted at time of delivery resulted in partial or complete amputation of the cervix. Both women recovered well postpartum.

**Conclusion:**

This rare phenomenon should be considered in the differential of intrapartum vaginal bleeding to avoid a missed or delayed diagnosis.

## 1. Introduction

Spontaneous cervical detachment is a rare complication reported in the literature as early as 1821 [1]. Although it has been described in the literature, in this new era of rising cesarean section rates and stricter adherence to progressive labor curves reports of spontaneous detachment have become a seemingly historic anecdote. However, we describe the presentation of a complete annular detachment in a multiparous woman in the setting of a failed trial of labor after cesarean delivery (TOLAC) without a significantly protracted labor course as well as a partial annular detachment in a multiparous woman 10 days following removal of an ultrasound-indicated cerclage.

## 2. Case 1

**Figure 1:**
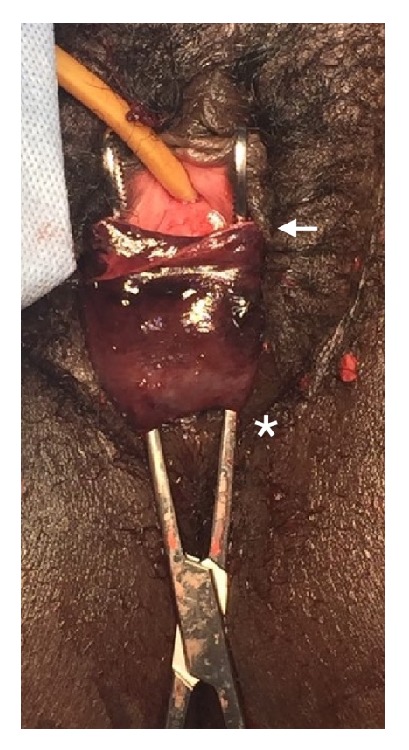
Intraoperative identification of cervical detachment of the necrotic anterior rim of the cervix in a complete annular detachment in a multiparous woman in the setting of a failed trial of labor after cesarean delivery. Asterisk indicates external os. Arrow indicates rim of anterior annular avulsion. Cervical canal canalized by ring forceps.

A 32-year-old multiparous woman was admitted at 37 2/7 weeks for induction of labor for abnormal antenatal testing. She had a history of a prior cesarean delivery for arrest of active labor at 7 cm and after extensive counseling chose to undergo a TOLAC. The initial cervical exam was 2 cm dilation and 50% effacement. Her labor was induced with oxytocin and amniotomy was performed at 5 cm dilation with clear fluid noted. She progressed from 2 cm to 5 cm over the course of 10 hours; interval time to progression to 6 cm was 3.5 hours. The patient then began to have a moderate amount of vaginal bleeding with associated minimal fetal heart rate variability and variable decelerations that did not improve with intrauterine resuscitation. She was taken to the operating room for repeat cesarean delivery due to concern for a uterine rupture and/or a placental abruption. Upon entry into the abdomen, there was no uterine rupture or dehiscence, nor was there evidence of a significant placental abruption. She delivered a male infant with a weight of 2659 g and Apgar scores of 9 and 9 at 1 and 5 minutes, respectively; arterial cord pH was 7.26. No extension of the hysterotomy was noted following delivery of the infant. Following repair of the hysterotomy, the bladder was noted to be distended and edematous, despite the presence of a patent Foley catheter. This finding prompted a vaginal exam to assist in evaluating the integrity of the lower uterine segment behind the bladder due to concern for an occult uterine rupture or dehiscence. The vaginal exam revealed a spontaneous detachment of the anterior rim of the cervix from approximately 9 to 3 o'clock. The avulsed portion of cervix appeared necrotic (Figure 1) and there was no bleeding noted at the site of cervical detachment. The cesarean delivery was completed, and an attempt was made to repair the site of the detachment vaginally, at which time the entirety of the cervix completely detached. No excessive bleeding was noted and placement of compression sutures at the site of detachment helped ensure hemostasis. Her recovery and postpartum course were otherwise unremarkable, and she was discharged on postoperative day #3. The patient was examined at a six-week postpartum visit. No cervix was seen on speculum exam and on digital exam the cervix was flush with the vaginal vault. The pathology report for the detached cervix showed diffuse hemorrhage and vascular congestion; there was no pathologic evidence of placental abruption.

## 3. Case 2

**Figure 2:**
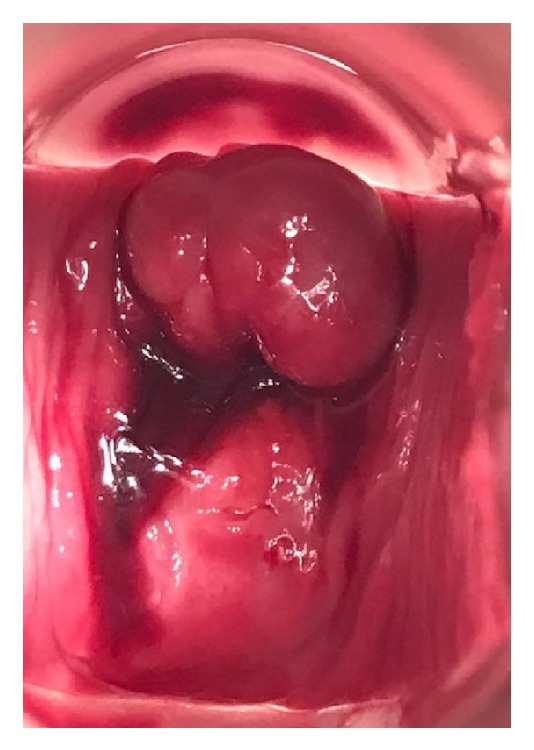
Six-week postoperative speculum evaluation of a multiparous woman who suffered a partial posterior cervical avulsion 10 days following removal of an ultrasound-indicated cerclage.

A 22-year-old multiparous woman with a history of primary sclerosing cholangitis complicated by portal hypertension, esophageal varices, and thrombocytopenia was admitted at 29 0/7 weeks for evaluation and management of shortness of breath and lower extremity edema. Her obstetric history was significant for prior spontaneous preterm birth with placement of an ultrasound-indicated McDonald cerclage at 20 1/7 weeks of gestation in the index pregnancy. At the time of cerclage placement, the 5 mm Mersilene (polyester) tape was noted to be suboptimally placed along the posterior rim, distal to the internal os, so a second McDonald cerclage of 0-polyester suture was placed cephalad to the tape. She was admitted at 28 0/7 weeks for preterm labor and the cerclages were removed with no remarkable anatomic irregularities. She received antenatal corticosteroids and magnesium sulfate during her admission for preterm labor and was discharged on hospital day #3 with a cervical exam of 1cm dilation and 70% effacement. During her readmission at 29 0/7 weeks for shortness of breath, she began to have contractions and moderate vaginal bleeding on hospital day #3. Her cervical exam was 4 cm and 90% effaced. She was transferred to labor and delivery, where she continued to have a rapid cervical change to 6 cm. A brief fetal heart rate bradycardia occurred with spontaneous recovery to a normal baseline. At this time, findings were concerning for placental abruption or possible coagulopathy from decompensated liver failure with a total estimated blood loss of 500 mL. Amniotomy was performed to expedite vaginal delivery. However, cervical dilation did not continue and complete cessation of vaginal bleeding was noted. Careful visualization of the cervix revealed that the posterior rim of cervix was detached and traversing the presenting fetal head creating a tension band that prevented further dilation or descent. This band of cervical tissue was clamped and suture-ligated followed by rapid delivery. She delivered a male infant with a weight of 1325 g and Apgar scores of 5 and 7 at 1 and 5 minutes, respectively; arterial cord pH was 7.33. Following delivery of the placenta, vaginal exam revealed a partial posterior cervical detachment from 2 to 9 o'clock. The avulsed portion of the cervix did not appear necrotic and was bleeding briskly. Hemostasis was achieved with a single running locked suture. Her postpartum course was otherwise unremarkable, and she was discharged on postpartum day #2. At her six-week postpartum visit her cervical exam was remarkable for a normal appearing anterior lip; however the posterior lip was not visible and not palpable on digital exam (Figure 2). Pathology was not available for the fragments of cervix that were excised and the placenta was remarkable for acute chorioamnionitis.

## 4. Discussion

Annular detachment of the cervix during labor is a rare complication and the exact incidence and mechanism are unknown. A series published in 1950 suggested that the incidence in a New York hospital was 1:11,000 deliveries; however, this series only had 5 reported cases [2]. The largest series published in 1947 is a review of 55 total cases worldwide [3]. During review of the series, the labor time was reported in a total of 41 cases, of which the average time spent in labor was approximately 58 hours with the longest being 108 hours [3]. While these case series described a likely underreporting of spontaneous cervical detachment during labor, the occurrence may in fact be rare in modern obstetrics due to clinical practice changes that now favor shorter labor lengths and more frequent intrapartum cervical exams than those reported in the antecedent literature.

Annular detachments are reported to be more common in primigravidae with prolonged labor [3]. Other predisposing factors described in the literature include age greater than 30 years old and mild cephalopelvic disproportion with a resulting transverse or posterior position of the infant's head. The detachment is thought to occur at 4-5 cm dilation and histologic examination usually notes hemorrhage, edema, congestion, and necrosis of the cervical tissue [4]. The exact mechanism for this complication is unknown; however many have theorized that compression of the cervix between the pubic symphysis and the fetal head may cause necrosis and edema at the site of the anterior lip of cervix [4]. However, posterior detachment has also been described [5]. Neri et al. suggest that previous cervical damage may contribute to the etiology of a posterior lip detachment of the uterine cervix. It was further theorized that pelvic examination with fingers might cause a hole in the edematous soft posterior lip of the cervix, and the detachment of the posterior lip may result from lateral extension of this hole [5].

Our first case is unique from previously reported cases in that the complication was noted in the setting of a failed trial of labor, confounding the cause of intrapartum vaginal bleeding and fetal heart rate decelerations. The vaginal bleeding experienced during labor was most likely secondary to the cervical detachment. However, because of the fetal heart rate decelerations in the setting of her history of a previous cesarean delivery and term stillbirth, overwhelming consideration was given to an evolving uterine rupture and/or placental abruption. At the time of cesarean delivery, neither a rupture nor an abruption was identified. The partial cervical detachment was only discovered after an edematous bladder prompted a vaginal exam to better assess the integrity of the lower uterine segment.

Our second case demonstrates another instance of occult cervical detachment. Despite cerclage removal 10 days prior to the onset of recurrent preterm labor, it is plausible that ischemia of the cervical tissue between the two cerclages may have resulted in mechanical compromise of the intervening tissue leading to decreased elasticity during cervical dilation. Initially, because of the concurrent fetal heart rate deceleration, moderate vaginal bleeding, and rapid preterm labor in a woman with liver disease, placental abruption and/or coagulopathy were suspected. However, cessation of bleeding following rupture of membranes was noted. As this is atypical of abruption or coagulopathy, careful digital and visual examination of the vagina and cervix performed and revealed partial cervical avulsion traversing the fetal head. The avulsed bucket-handle defect in the cervix was noted to be preventing continued cervical dilation. Ligation of this tension band facilitated rapid continued cervical dilation and delivery. While we do not recommend expectant management of cervical detachment in anticipation of vaginal delivery for every patient, our second case demonstrates that this course of action may prevent needless cesarean delivery if delivery is imminent.

Both women received counseling postpartum on the implications of their cervical detachments and absence of a complete cervix for future pregnancies. There have been several reported cases of subsequent vaginal deliveries without cervical insufficiency or recurrent abortions [3]. However, given the complete absence of cervical tissue in the first case and history of recurrent preterm delivery with subsequent detachment of >50% of her cervix in the second case, our patients' risk of cervical insufficiency in subsequent pregnancies is likely high. We believe consideration may be given to offering an abdominal cerclage prior to future pregnancies.

## Additional Points

*Teaching Points*. (1) Partial or complete cervical detachment may be considered as a rare cause of intrapartum vaginal bleeding and may be accompanied by fetal heart rate decelerations. (2) Apart from the common intrapartum occurrences of vaginal bleeding and fetal heart rate decelerations, the identification of a partial or complete cervical avulsion requires physician vigilance and careful physical examination. (3) A partial cervical avulsion forming a bucket-handle abnormality can impede further cervical dilation and mimic an arrest of dilation.
